# The COVID-19 Pandemic Enhanced the Decade-Long Trend of the Decreasing Utilization of Antibiotics

**DOI:** 10.3390/antibiotics12050927

**Published:** 2023-05-18

**Authors:** Christian Tanislav, Josef Rosenbauer, Karel Kostev

**Affiliations:** 1 Department of Geriatrics and Neurology, Diakonie Hospital Jung Stilling Siegen, Wichernstrasse 40, 57074 Siegen, Germany; 2 Epidemiology, IQVIA, 60549 Frankfurt am Main, Germany

**Keywords:** antibiotics, infections, COVID-19 pandemic

## Abstract

Purpose/Introduction: A decline in antibiotic (AB) prescriptions was reported during the coronavirus 2019 (COVID-19) pandemic. Therefore, we investigated AB utilization during the COVID-19 pandemic using data from a large database in Germany. Methods: AB prescriptions in the Disease Analyzer database (IQVIA) were analyzed for each year between 2011 and 2021. Descriptive statistics were used to assess developments in relation to age group, sex, and antibacterial substances. Infection incidence rates were also investigated. Results: In total, 1,165,642 patients received antibiotic prescriptions during the entire study period (mean age: 51.8; SD: 18.4 years; 55.3% females). AB prescriptions started to decline in 2015 (505 patients per practice), and this development persisted until 2021 (2020: 300 patients per practice and 2021: 266 patients per practice). The sharpest drop was observed in 2020 and occurred in both women and men (27.4% and 30.1%). In the youngest age group (≤30), the decrease was −56%, while in the age group >70, it was −38%. The number of patients with prescriptions for fluoroquinolones dropped the most, falling from 117 in 2015 to 35 in 2021 (−70%), followed by macrolides (−56%) and tetracyclines (−56%). In 2021, 46% fewer patients were diagnosed with acute lower respiratory infections, 19% fewer with chronic lower respiratory diseases, and just 10% fewer with diseases of the urinary system. Conclusion: AB prescriptions decreased more in the first year (2020) of the COVID-19 pandemic than infectious diseases did. While the factor of older age influenced this trend negatively, it remained unaffected by the factor of sex and the selected antibacterial substance.

## 1. Introduction

In the last decades, the overuse and misuse of antibiotics has led to an increase in antimicrobial resistance [1,2]. Mutual influencing factors which are not limited to national borders sustain and aggravate this development [2]. The most noteworthy of these factors include international travel, migration between countries, and eventually physicians and patients handling antimicrobial drugs [3,4,5,6,7,8,9]. Increasing antimicrobial resistance has become a safety concern, especially in multimorbid patients and in those receiving treatment in intensive care units [10,11]. Of all the factors responsible for this development, the initial point at which to prescribe antibiotics might be the most influential [1,12]. Educational programs focusing on the appropriate use of antibiotics have proven effective in the past, with the permanent implementation of such programs in clinical algorithms often becoming standard practice [1,7,12]. As indicated in previous investigations, antibiotic usage patterns rapidly returned to the status they held prior to the implementation of a program when antimicrobial stewardships were discontinued [7]. An increase in antibiotic usage has been noted in recent decades, and this trend varyies on a regional basis [13]. A higher level of antibiotic use in high-income countries, such as those in Europe, has remained stable, with a slight tendency to decrease over the last few decades. Rates have increased dramatically in regions such as the Middle East and North Africa, drawing level with 2020 values observed in Europe [13]. From 2020 onwards, however, a global pandemic caused by severe acute respiratory syndrome coronavirus 2 (SARS-CoV-2) had drastic socioeconomic effects and dramatic consequences regarding the utilization of medical care resources and the treatment of diseases [14,15]. Due to a general behavioral change resulting from the implementation of strict hygiene rules, a decrease in rates of non-COVID-19 infectious diseases occurred [16,17,18,19,20]. As previously reported, a dramatic decrease in non-COVID-19-related infections (upper respiratory tract infections: −36%; gastrointestinal infections: −44%; and urinary tract infections: −11%) was described [20]. Based on this observation, a decrease in the utilization of antibiotics could also be expected. In a study investigating the use of antibiotics during the COVID-19 pandemic in hospitals in China compared to previous rates, a remarkable decrease was noted [21]. In this context, Akmatov and colleagues investigated prescriptions for antibiotics in children (≤14 years) in Germany, detecting an annual decrease of 6% prior to the pandemic, with the figure halving (57%) in 2021 in comparison to the level in 2019 [22].

It is for this reason that we aimed to investigate the trend of antibiotic utilization prior to the COVID-19 pandemic and during the COVID-19 pandemic, using data from a large database supplied with data by general practitioners and pediatricians in Germany.

## 2. Methods

This retrospective cross-sectional study used data from the Disease Analyzer database (IQVIA), full details of which have been published elsewhere [23]. The Disease Analyzer database is composed of sociodemographic, diagnosis, and prescription data obtained from general and specialized practices in Germany. Although this database covers only approximately 3–4% of the private practices in Germany, it has previously been demonstrated to be representative and has been widely used for epidemiological studies in recent years [23,24,25].

All individuals (*n* = 4,175,400) with at least one visit to one of 477 general practices (GP) across Germany represented in the database between January 2011 and December 2021 were included. Each practice delivered data continuously between 2011 and 2021. The study outcome was the number of patients with at least one antibiotic (ATC: J01) prescription per practice in each year between 2011 and 2021. These analyses were performed separately for four age groups (≤30, 31–50, 51–70, and >70 years) and separately for women and men. In addition, the antibiotic drug classes found in the database, including penicillins, tetracyclines, cephalosporins, trimethoprims, macrolides, and fluoroquinolones, were shown separately. To understand the possible reasons for the trends found in the study, we performed two post hoc analyses. First, the prevalence of antibiotic prescriptions was calculated for the years 2011, 2016, and 2021 as the number of patients with an antibiotic prescription divided by the total number of patients who had visited their GP in each year. Second, the number of patients with each of the four most common diagnoses listed as prescription diagnoses in 2011–2021 were evaluated and displayed. These diagnoses included acute upper respiratory infections (ICD-10: J00–J06), other acute lower respiratory infections (ICD-10: J20–J22, here, mainly acute bronchitis), chronic lower respiratory diseases (ICD-10: J40–J47, here, mainly chronic bronchitis), and other diseases of the urinary system (ICD-10: N30–N39, here, mainly lower urinary tract infections).

This study used descriptive statistics. Due to the very large counts involved, each small difference would become significant, and differences between the periods were therefore not assessed using statistical tests. Analyses were carried out using SAS version 9.4 (SAS Institute Inc: Cary, NC, USA).

## 3. Results

**Figure 1:**
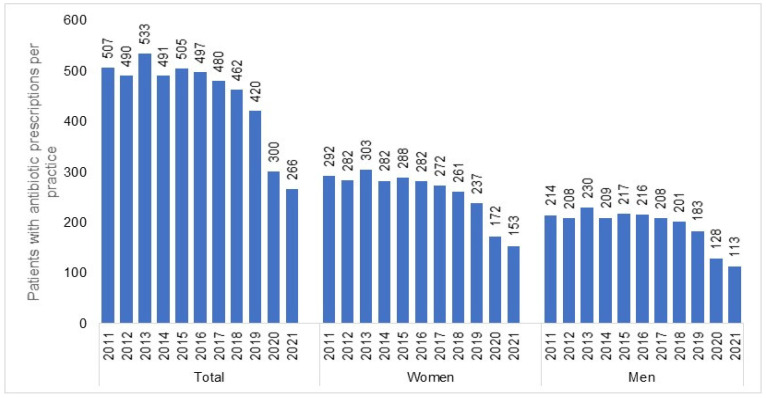
Number of patients (total, women, and men) with at least one antibiotic prescription per practice in 2012–2021.

**Figure 2:**
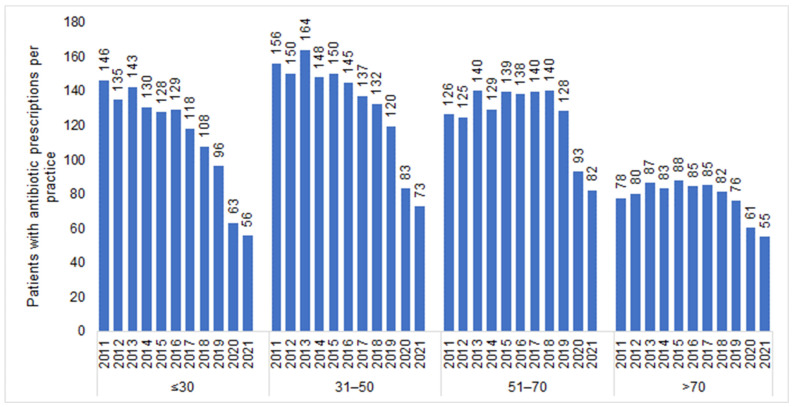
Number of patients with at least one antibiotic prescription per practice in 2012–2021 by age group.

The number of individuals who visited one of the 477 GP practices included in our study increased from 1,085,418 in 2011 to 1,442,999 in 2021. In total, 1,165,642 patients received antibiotic prescriptions throughout the entire study period (mean age: 51.8; standard deviation: 18.4 years; 55.3% females). In all years investigated, women received more AB prescriptions than men (e.g., 2011: 292 prescriptions per practice for women versus 214 prescriptions per practice for men; 2021: 153 prescriptions per practice for women versus 113 prescriptions per practice for men). We observed a negative trend in the number of patients with an AB prescription (Figure 1), starting in 2015 (505 patients per practice) and persisting until 2021 with the most significant decreases recorded in 2020 (300 patients per practice) and 2021 (266 patients per practice) (Table 1). This sharp drop was observed in both women and men (Figure 1) and across all age groups (Figure 2). However, while (≤30) the decrease in the youngest age group was from 128 patients per practice in 2015 to 56 patients per practice in 2021 (−56%), the decline in the age group >70 was weaker, dropping from 88 patients per practice in 2015 to 55 patients per practice in 2021 (−38%) (Figure 2).

**Figure 3:**
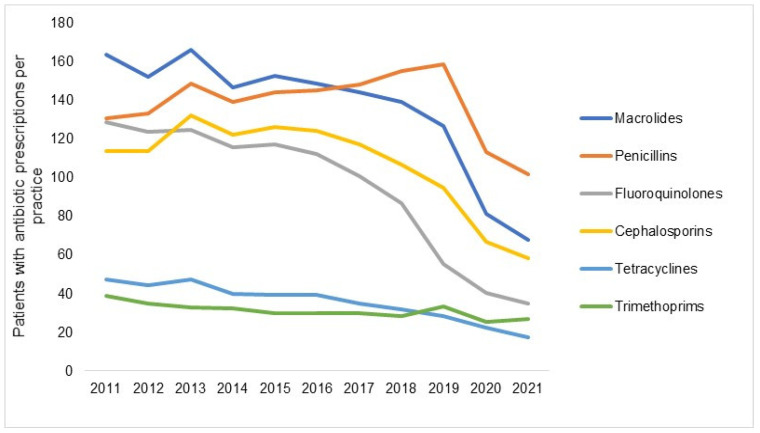
Number of patients with at least one antibiotic prescription per practice in 2012–2021 by antibiotic drug class.

**Figure 4:**
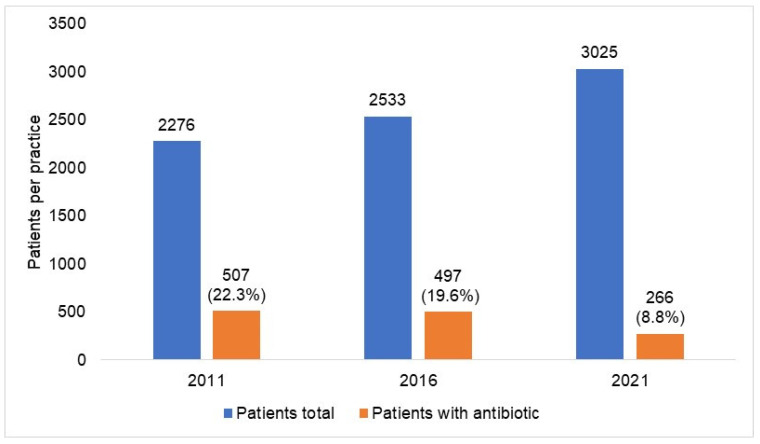
Prevalence of antibiotic prescription in 477 general practices in 2011, 2016, and 2021. In general, from 2011 to 2021, the absolute frequency of patients treated per practice increased from 2276 to 3025, and the percentage of those treated with antibiotics decreased from 22.3% to 8.8%.

Figure 3 shows the number of patients with at least one antibiotic prescription per practice in 2012–2021 by antibiotic drug classes. Although a decrease was observed for each AB drug class, it differed in expression depending on the respective class. The decrease was the greatest among patients with prescriptions for fluoroquinolones, with the figure changing from 117 in 2015 to 35 in 2021 (−70%), followed by macrolides (−56%) and tetracyclines (−56%), while the decrease in trimethoprim prescriptions, which were rarely prescribed, amounted to just 10% and was only observed in 2020 and 2021 and not in previous years (Figure 3). Figure 4 shows the prevalence of AB prescriptions in 2011 (22.3%), 2016 (19.6%), and 2021 (8.8%).

**Figure 5:**
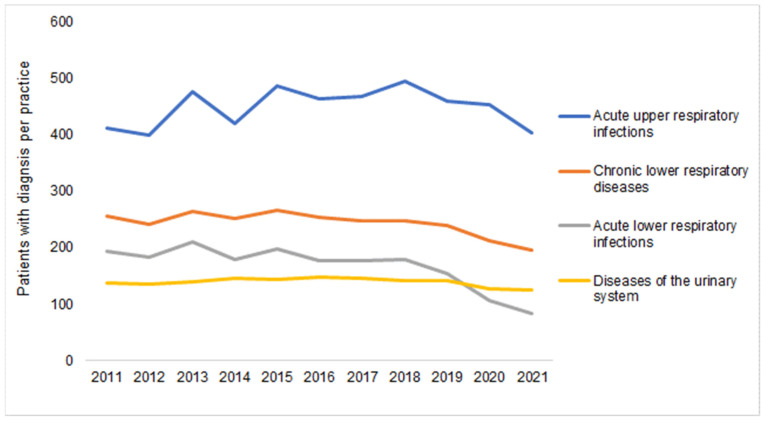
Number of patients with at least one infectious disease documented per practice in 2012–2021.

Finally, Figure 5 shows the number of patient diagnoses for one of the diseases which are often listed as a reason for an AB prescription. No clearer negative trend could be seen between 2011 and 2019 (Table 1), with the number of diagnoses only decreasing in 2020 and 2021 compared to 2019. For example, in 2021, 46% fewer patients were diagnosed with acute lower respiratory infections, 19% fewer with chronic lower respiratory diseases, and just 10% fewer with diseases of the urinary system (Figure 5).

**Figure 6:**
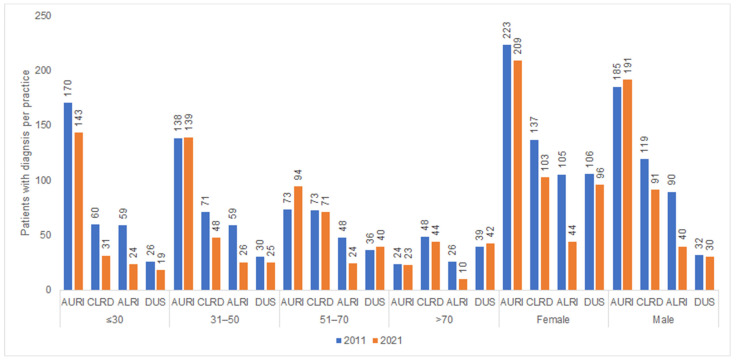
Number of patients with at least one infectious disease documented per practice in 2011 and 2021 by age and sex.

Figure 6 shows the number of patient diagnoses for one of the diseases which are often listed as a reason for an AB prescription in 2011 and 2021 by sex and age. Women were diagnosed with acute lower respiratory tract infections and infectious diseases of the urinary system more frequently than men.

AURI—acute upper respiratory infections; CLRD—chronic lower respiratory diseases; ALRI—acute lower respiratory infections; DUS—diseases of the urinary system.

## 4. Discussion

Our study indicated a decreasing trend in rates of AB prescriptions starting in 2015 and culminating in 2021. The two years of the COVID-19 pandemic (2020 and 2021) accelerated this development dramatically. In contrast, the incidence rates for infectious diseases remained constant prior to the pandemic, with a decreasing trend only observed in 2020 and 2021. However, the decline observed here was not significant and does not fully explain the decrease in the number of AB prescriptions. The trend depicted was evident, although the absolute number of patients in the selected practices increased over time, even during the pandemic period. The decline in AB prescriptions detected in our study applies to all AB classes and seems to be weaker in the subgroup of patients older than 70 years of age. In general, the utilization of antibiotics was slightly higher in female patients than in male patients.

The decline in prescriptions of antibiotics was already evident in advance of the COVID-19 pandemic. In 2020, Holstiege et al. reported a decrease of 10–50% in antibiotic prescriptions nationwide in Germany between 2010 and 2018, with a tendency to avoid prescribing antibiotics amongst individuals of a younger age [26,27]. The authors attributed this development to the numerous initiatives that have been implemented to reinforce the appropriate use of antibiotics in Germany [26,27]. A similar trend was obvious in our study; we detected a decrease of 15% from 2011 to 2018 in the age group 31–50 years. While this observation could be regarded generally as an amelioration in attitudes to therapy with antibiotics, the decline of 28.6% in AB prescriptions observed in 2020 and 2021 remains a topic of debate. It could be speculated that the trend observed prior to the pandemic, with yearly decreases of 1.6–9.1% in AB prescription rates, might have had some effect during the pandemic as well; however, in comparison to 2019, the decrease observed in 2021 falls out of line, indicating that a number of other factors influenced the development observed during this period. Nor can the incidence rates of infectious diseases fully explain the profound reduction in AB prescriptions between 2019 and 2021. While the number of AB prescriptions declined by 28.6% during this period, the incidence rate of infectious diseases decreased by 9.6% in the same period. At this point, we need to take into account the fact that when we analyzed the data, we considered those disorders that most likely required antibiotics, meaning that some conditions may have been missed. Interestingly, the decline observed from 2020 to 2021 (−11.3%) remains within the range of the decreases observed prior to the pandemic, which supports the hypothesis of a collapse in AB prescriptions from 2019 to 2020 and a normalization a year later at a level comparable to the pre-pandemic level. Akmatov and colleagues also observed this effect when they investigated the development of AB prescriptions in children, identifying a dramatic decline from 2019 to 2020 with a slight increase in 2021 [22]. However, regardless of the extent to which the pre-pandemic development and the decline in the incidence of infectious diseases might have influenced the profound reduction in AB prescriptions in 2020, there are a number of other factors that could be responsible for the development observed. It could also be speculated that the factors described above, such as limited access to medical care and changes in individual care-seeking behaviors during the pandemic, are responsible for the marked decline in AB prescriptions in 2021 that was detected in our study [28,29,30,31,32,33].

The decrease in the prescription of antibiotics during the first year (2020) of the COVID-19 pandemic had different impacts among the various age categories. As previously reported, we identified the greatest reduction in AB prescriptions in the younger age classes, while the reduction was less pronounced in patients aged ≥70 years [32,33]. The prescription of antibiotics is determined in particular by the judgment of the physician, who might consider current recommendations as well as the presumed severity of the infection and the need for treatment. While recommendations for antibacterial therapy persisted despite developments during the pandemic, the perception of the necessity of such treatment was obviously different in the environment of the pandemic. It can be speculated that in 2020, physicians were more inclined to tolerate infections without antibacterial treatment in younger individuals, while this was less the case in older adults. This behavior could be explained to some extent by the increasing general morbidity in older age groups, which have a higher vulnerability with respect to exposure to infection [34,35]. However, there is no definitive or conclusive explanation for this observation.

In line with other investigations, our study found that the decrease in AB prescriptions in 2020 applied across all antibiotic classes [32]. Our findings indicate that the substance selected for the therapy is of minor relevance when circumstances such as the COVID-19 pandemic might influence the physician’s behavior. Similarly, the sex factor seemed to not influence the decline in AB prescriptions from 2019 to 2020 (females: −27.4% versus males: 30.1%), although the subgroup of females comprised slightly more individuals than the corresponding subgroup of males (female participants: 55.3% versus male participants: 44.7%).

The present study was based on analyses of more than one million patients per year over seven years and combines prescriptions and diagnoses. Nonetheless, the study was also subject to several limitations that should be acknowledged at this point. First, no information was available on the severity of infections, although there may be a positive relationship between the severity of the disease and the prescription of antibiotics. Second, we cannot exclude the possibility that diagnoses were sometimes misclassified or that their coding was missing within the ICD-10 coding system. Third, this study used data from GP practices and did not include any data from specialists or hospitals. Finally, we cannot investigate the role of antibiotics on the gut microbiome. For example, Yeoh et al. reported that dysbiosis of the gut microbiota following the resolution of COVID-19 could contribute to post-COVID-19 syndrome [36].

## 5. Conclusions

Our study identified a marked reduction in outpatient antibiotic prescriptions during the first year (2020) of the COVID-19 pandemic, which returned to pre-pandemic levels in 2021. This trend was observed despite the increase in the number of consultations per practice over the period investigated. The decline in infections during this period does not fully explain the drop in AB prescriptions in 2020. Physicians may have changed their prescribing behavior in this period; factors such as sex and the specific antibacterial substance prescribed did not influence this behavior, while the factor of older age seemed to lessen the reluctance to prescribe ABs in the environment of the pandemic. In summary, we appreciate the change in individual behaviors resulting in a patient’s refrain from seeking medical care and the reduction in the incidence of non-COVID-19 infectious diseases during the pandemic, which are the most important determinants for the findings depicted in our study.

## Figures and Tables

**Table 1:** Yearly absolute reduction in antibiotic prescription prevalence (absolute values and percentages).

	Yearly Difference					
Patients with Antibiotic Prescriptions	2016–2015	2017–2016	2018–2017	2019–2018	2020–2019	2021–2020
Absolute number of patients per practice	497−505 = −8	480−497 = −17	462−480 = −18	420−462 = −42	300−420 = −120	266−300 = −34
Percentage reduction	−1.6%	−3.4%	−3.8%	−9.1%	−28.6%	−11.3%
Infectious diseases *	2016−2015	2017−2016	2018−2017	2019−2018	2020−2019	2021−2020
Absolute number of patients per practice	1044−1094 = −51	1039−1044 = −4	1065−1039 = +26	997−1065 = −98	901−997 = −96	810−901 = −91
Percentage reduction	−4.6%	−0.4%	2.5%	−6.4%	−9.6%	−10.1%

* Diseases considered include acute lower and upper respiratory tract infections, chronic lower respiratory diseases, and infectious diseases of the urinary system.

## Data Availability

Anonymized raw data are available upon reasonable request by contacting the corresponding author.
